# A feedback loop between cell proliferation and ROS regulates ferroptosis sensitivity

**DOI:** 10.3389/fcell.2026.1756238

**Published:** 2026-03-25

**Authors:** Eric Seidel, E. Yaren Itak, Fabienne Müller, F. Isil Yapici, Johannes Brägelmann, Johannes Berg, Silvia von Karstedt

**Affiliations:** 1 University of Cologne, Faculty of Medicine and University Hospital Cologne, Department of Translational Genomics, Cologne, Germany; 2 University of Cologne, Faculty of Medicine and University Hospital Cologne, CECAD Cluster of Excellence, Cologne, Germany; 3 Institute for Biological Physics, University of Cologne, Cologne, Germany; 4 University of Cologne, Faculty of Medicine and University Hospital Cologne, Center for Molecular Medicine Cologne, Cologne, Germany; 5 University of Cologne, Faculty of Medicine and University Hospital Cologne, Mildred Scheel School of Oncology (MSSO), Cologne, Germany

**Keywords:** feeedback loop, ferroptosis, lipid ROS, modelling, ROS

## Abstract

**Background:**

Ferroptosis is a form of regulated cell death characterized by iron-dependent lipid peroxidation and membrane rupture. While cellular populations reaching confluence are known to have limited sensitivity to ferroptosis, an understanding of the interplay between growth dynamics, reactive oxygen species (ROS) levels, metabolism and ferroptosis is currently lacking. This study aimed to establish a regulatory framework for the systemic interplay of these biological processes.

**Results:**

Here we use live-cell imaging coupled to ROS tracing to reveal a feedback loop between population growth and ferroptotic cell death. Starting out from the observation that the cellular proliferation rate declines with increased cellular density, we find that ROS levels also decline with increasing cellular density. In turn, low ROS levels make cells insensitive to ferroptosis, which enables population growth. Conversely, keeping cell numbers and drug concentration/cell constant while restricting growth space led to reduced proliferation, reduced ROS and decreased ferroptotic cell death. We find that this feedback between population growth and ferroptotic cell death leads to two steady states: (i) a ferroptosis-insensitive state characterized by slow growth, low levels of ROS and low rates of cell death and (ii) a ferroptosis-sensitive state characterized by rapid growth, ROS accumulation, and high rates of ferroptosis. A mathematical model of the feedback mechanism predicts the long-term fate of populations as well as their ferroptosis sensitivity when external conditions impacting cell proliferation rates, ROS, or both are changed. We tested the proposed feedback mechanism experimentally by interfering with lipid hydroperoxide clearance and by increasing cellular and lipid ROS production through a galactose-promoted OXPHOS switch.

**Conclusion:**

We find a feedback loop between population growth and ferroptotic cell death that dictates cellular fate (growth or cell death via ferroptosis) and is mechanistically determined by the levels of metabolic ROS. These results provide a unifying framework that dynamically links population growth and metabolic ROS regulation with ferroptosis sensitivity.

## Background

The observation that cysteine removal kills cells in culture only under low density dates back to the establishment of human cell culture by Harry Eagle in the 1960ies (Eagle, 1960). We now know that in cell culture cysteine removal induces ferroptosis, an iron-dependent type of regulated necrosis (Gao et al., 2018). Ferroptotic cells present with loss of plasma membrane integrity due to excessive lipid peroxidation, osmotic cytoplasmic swelling, and mitochondrial fragmentation and failure (Dixon et al., 2012; Friedmann Angeli et al., 2014; Riegman et al., 2020). Glutathione peroxidase 4 (GPX4) constitutively hydrolyses lipid hydroperoxides thereby preventing ferroptosis (Yang et al., 2014). Consequently, genetic or pharmacological inactivation of GPX4 induces ferroptotic cell death (Friedmann Angeli et al., 2014; Seiler et al., 2008; Yang et al., 2014). In recent years, several other cellular ferroptosis defense mechanisms apart from GPX4 have been described, including the ferroptosis suppressor protein 1 (FSP1) pathway, the Nuclear factor erythroid 2-related factor 2 (NRF2)-regulated axis, receptor-mediated uptake of iron and selenoprotein P (SEPP1) and changes in iron metabolism and polyunsaturated fatty acid (PUFA)-synthesis (all reviewed in detail recently (Dai et al., 2025; Dixon and Olzmann, 2024)). One of the most defining hallmarks of ferroptotic cell death to date is excessive membrane lipid peroxidation driven by lipid reactive oxygen species (lipid ROS) which unlike general ROS reside within the plasma- and other cellular membranes (Kagan et al., 2017; Wiernicki et al., 2020). Lipid peroxidation during ferroptosis was recently shown to be initiated at the endoplasmic reticulum (ER) membrane and subsequently spreads to and accumulates at the plasma membrane (von Krusenstiern et al., 2023). This then leads to the collapse of transmembrane cation gradients, which, in turn, may ultimately cause nanopore formation and membrane rupture and cell lysis (Dixon and Olzmann, 2024; Hirata et al., 2023). In cycling cells mitochondria are a major source of ROS as a by-product of oxidative phosphorylation (OXPHOS) (Ivanova et al., 2021), which in the presence of protein iron-sulphur clusters can give rise to lipid ROS (Homma et al., 2021). Thereby, mitochondrial OXPHOS is thought to indirectly feed into ferroptosis susceptibility of cells. Notably, cells generating cellular ATP mainly through glycolysis—the Warburg effect—also generate less ROS (Martens et al., 2005), which may influence ferroptosis sensitivity (Homma et al., 2021). While apoptosis sensitivity is either promoted or inhibited by increasing cellular population density depending on specific cell type (Hannah et al., 1998; Kühn et al., 1999; Vultur et al., 2004), increasing density has been observed to restrict the capability of cells to undergo ferroptotic cell death (Schneider et al., 2010; Seiler et al., 2008). Herein, E-cadherin on epithelial cells was shown to prevent YAP1-mediated induction of two mediators promoting ferroptosis sensitivity ACSL4 and TFRC (Wu et al., 2019). Yet, similar observations have also been made for mesenchymal cells lacking expression of E-cadherin suggesting additional mechanisms by which high cellular confluence restricts ferroptotic cell death (reviewed in (Vucetic et al., 2020)). Indeed, high cellular density was shown to downregulate ROS and ferric iron content (Yan et al., 2024), known outputs and inputs, respectively, of mitochondrial OXPHOS. While the mitochondrial tricarboxylic acid (TCA) cycle was shown to promote ferroptosis (Gao et al., 2018), it is only poorly understood how cellular confluence, cellular metabolic state, cellular ROS levels and ferroptosis sensitivity might be connected. Understanding this connection, however, will likely reveal targetable growth states in cellular malignancies in which proliferation and/or metabolism is deregulated such as cancer. Here, we used a combined approach of cellular experimental work with mathematical modelling to address this connection.

## Materials and methods

### Cells

Mouse embryonic fibroblasts (MEFs) were kept in T-75 flasks at 37 °C with 5% CO_2_ and all media were supplemented with 10% FCS and 1% P/S. MEFs were tested for *mycoplasma* at regular intervals (*mycoplasma* barcodes, Eurofins Genomics) (Table 1). Cells were passaged every 3-4 days and discarded after 3 weeks.

**TABLE 1:** Reagents and resources used in this study.

Reagent or resource	Suppplier	Cat#
Chemicals		
BODIPY C11	Thermo Fischer	D3861
CellRox Green	Thermo Fischer	C10444
DMEM, high glucose, GlutaMAX™ supplement	Thermo Fisher	61965059
DMEM, glucose free, GlutaMAX™ supplement	Thermo Fisher	11966025
Dimethyl sulfoxide (DMSO)	PAN Biotech	P60-36720100
Blasticidn	Thermo Fisher	15205
DRAQ7	Biolegend	424001
FCS	Sigma-Aldrich	F0804
Ferrostatin-1	Cayman Chemicals	17729
Glucose monohydrate	Merck	1.04074.0500
Galactose	Sigma-Aldrich	G5388
ITS+	Corning	354352
PBS, pH 7.4	Thermo Fisher	10010056
Penicillium Streptomycin (PS)	Sigma	P4333-100ML
RSL3	Selleck Chem	S8155
STY-BODIPY	Cayman Chemical	Cay27089-500
Trypsin	Serva	9002-07-7
Kits		
NucleoSpin RNA kit	Macherey-Nagel	740955250
Lexogen QuantSeq kit	Lexogen Austria	166.96
Data deposition		
Bulk RNA seq	​	Available from the corresponding author without undue reservation: svonkars@uni-koeln.de
Cell lines		
KRAS wild type expressing “Rasless” mouse embryonic fibroblasts (MEFs)	RAS Initiative at the Frederick National Laboratory for Cancer Research (FNLCR), US	N/A
Software and algorithms		
nf-core suite (v3.7)	Ewels et al. (2020)	https://github.com/nf-core/rnaseq/releases/tag/3.7
STAR (v2.7.10a)	Dobin et al. (2013)	https://github.com/STAR-Fusion/STAR-Fusion/wiki/STAR-Fusion-release-and-CTAT-Genome-Lib-Compatibility-Matrix
Salmon (v1.5.2)	Patro et al. (2017)	https://github.com/COMBINE-lab/salmon/releases/tag/v1.5.2
DESeq2 (v1.36.0)	Love et al. (2014)	https://bioconductor.org/packages/release/bioc/html/DESeq2.html
fdrtool (v1.2.17)	Strimmer (2008)	https://cran.r-project.org/web/packages/fdrtool/index.html
gprofiler2 (v0.2.2)	Kolberg et al. (2020)	https://cran.r-project.org/web/packages/gprofiler2/index.html
Flowjo version 10.6.2	BD Life Sciences	https://www.flowjo.com/solutions/flowjo/downloads/previous-versions
Prism version 10	GraphPad	https://www.graphpad.com
IncuCyte 2024B	Sartorius	https://www.sartorius.com/en/products/live-cell-imaging-analysis/live-cell-analysis-software

### Quantification and statistical analysis

Raw data from bulk RNA sequencing was processed as described below, and all other raw data was processed using Excel and GraphPad prism. For statistical testing and generation of figures, GraphPad Prism 10 was used. Two-tailed t-tests were used to compare two conditions, and two-way ANOVA was used to compare multiple samples. All measurements were performed at least three times if not stated otherwise, and results are presented as mean ± standard error mean (SEM). ns: not significant; ∗p < 0.05; ∗∗, p < 0.01; ∗∗∗, p < 0.001; ∗∗∗∗, p < 0.0001.

### Population doublings

Cells were seeded in T25-flasks (LD: 329.000; MD: 724.000; HD: 1.578.000 cells per flask) in 5 mL medium and incubated for 24, 48 and 72 h. Afterwards, cells were detached in 1 mL trypsin+ 5 mL medium as described above and counted using a Casy Model TT cell counter (Roche Diagnostics). Population doublings were then calculated using the following equation:

$$Population\;doublings=\log_{2}\left(cells\;counted\right)-\log_{2}\left(cells\;seeded\right)$$

### Reagents

Stock solutions were prepared as follows: RSL3 was dissolved in DMSO at 1 mM, and Ferrostatin-1 was dissolved in DMSO at 10 mM. All other reagent-related information is found in Table 1.

### Treatments

For treatment with RSL3, cells were seeded in 24-well plates at 25.000 (low density; LD), 55.000 (medium density; MD) or 120.000 (high density; HD) per well. After 24 h, RSL3 was added to the medium at a final concentration of 0.1 µM. For glucose/galactose exchange experiments, cells were seeded in DMEM GlutaMAX™ supplemented with PS and FCS as described above. After 24 h, cells were washed thrice with PBS (Gibco) and received glucose-free DMEM (Gibco) supplemented with either 4,5 g/L glucose or galactose, 4 μg/mL blasticidin, 1% P/S and 1% Insulin-Selenium-Transferrin+ (ITS+) supplement (Corning).

### RNA sequencing

For RNA sequencing, MEFs were plated in a 6 well plate at 125.000 (LD) or 600.000 (HD) cells per well. After 24 h, cells were washed with PBS and RNA isolation was done using the NucleoSpin RNA kit (Macherey-Nagel). cDNA libraries amplified from the 3′UTR were generated from total RNA using the Lexogen QuantSeq kit (Lexogen, Austria) according to the standard protocol and sequenced with a 50-bp single-end protocol on Illumina HiSeq4000 sequencer (Illumina, United States).

Primary data analysis was conducted using the RNA-seq pipeline from the nf-core suite (v3.7) (Ewels et al., 2020); sequencing reads were aligned to the GRCh38 (v103) human reference genome using STAR (v2.7.10a) (Dobin et al., 2013). Gene quantification was conducted using Salmon (v1.5.2) (Patro et al., 2017). The pipeline was executed with default parameters. Downstream differential expression analysis was performed using DESeq2 (v1.36.0) (Love et al., 2014), with default parameters. To enhance the accuracy of fold-change estimation, we included mouse ID as a batch effect in the design matrix. For some comparison, the original p-values inferred by DESeq2 revealed significant deviation from the expected uniform null distribution, suggesting low sensitivity. To correct for this, we recomputed the raw p-values using fdrtool (v1.2.17) (Strimmer, 2008) to increase the power of the differential expression procedure while maintaining efficient control for false discovery. Subsequently, the Benjamini–Hochberg procedure was applied to correct the p-values for multiple tests.

GO enrichment analysis was conducted using gprofiler2 (v0.2.2) (Kolberg et al., 2020). The selection criteria focused on differentially expressed genes, as defined above. Using ordered gene query and gProfiler’s “g_SCS” method for p-value adjustment, which accounts for the hierarchical structure of GO terms, enriched GO terms were identified among the differentially expressed genes.

### Flow cytometry

MEFs were seeded in 24 or 12 well plates, treated after 24 with RSL3 as described above and incubated for the indicated durations. BODIPY C11 was added to each well at a final concentration of 5 µM followed by incubation for 30 min at 37 °C. Cells were washed, detached and the cell pellet was then resuspended in 200 μL of PBS with 2% FCS and [1 mg/mL] propidium iodide (PI) (Table 1). CellROX was added to each well at a final concentration of 2.5 µM followed by incubation for 30 min at 37 °C. Cells were washed, detached and the cell pellet was then resuspended in 200 μL of PBS with 2% FCS and [1 mg/mL] propidium iodide (PI). Flow cytometry data were acquired on the BD LSRFortessa (BD Biosciences) and analyzed with Flowjo V10.6.2.

### Live cell imaging (IncuCyte)

MEFs were plated in 12- and 24-well plates and treated as indicated. For dead cell quantification DRAQ7 [0.3 µM] was used. For lipid ROS quantification STY-BODIPY [1 µM] was used. Cells were imaged every 2 h using the 10× objective within the IncuCyte SX5 live cell imaging system (Sartorius). Analysis for confluence, DRAQ7-positive (dead), reduced- and oxidized-BODIPY positive cells was performed using the Software IncuCyte 2024B (Sartorius). In order to account for different starting densities, dead cell counts were normalized to confluency as previously described (Roeck et al., 2025; Szalai and Engedal, 2018; Yapici et al., 2025), and lipid ROS was normalized by calculating the ratio of oxidized to reduced STY-BODIPY as reported previously (Yapici et al., 2025). To quantify the number of live cells (live cell confluence) and their lipid ROS levels we carried out additional image analysis to estimate the fraction of the Incucyte signal contributed from dead cells and from living cells. We converted all video frames and images per vessel and applied a color mask to exclude dead cells based on their DRAQ7 signal. Exclusion of the signal from dead cells was performed by expanding the dead cell mask and identifying its overlap with the fluorescence and coverage mask, see Supplementary Document S1.

### Cellular lipid ROS quantification

To quantify the level of cellular lipid ROS (lipid ROS levels averaged over live cells) we normalized the lipid ROS signal from live cells by dividing by the live cell confluence.

### Mathematical model and data fitting

The first differential equation in Figure 2B gives the rate of change of cell numbers relative to 100% confluence (left hand side) as a balance between the rates of cell divisions and cell death, respectively (right hand side). Analogously, the second equation gives the rate of change of lipid ROS levels (left hand side) and lipid ROS generation and clearance rates (right hand side). We estimate the model parameters by fitting the solution of the differential equations in Figure 2B to the data using a weighted least-square fit. These parameter estimates give the fits to the time courses in Figures 4E,F,H,I and the basins of attraction in Figures 4G,J. Details are given in Supplementary Document S1.

## Results

### Population growth dynamics are linked with cellular ROS levels

**FIGURE 1:**
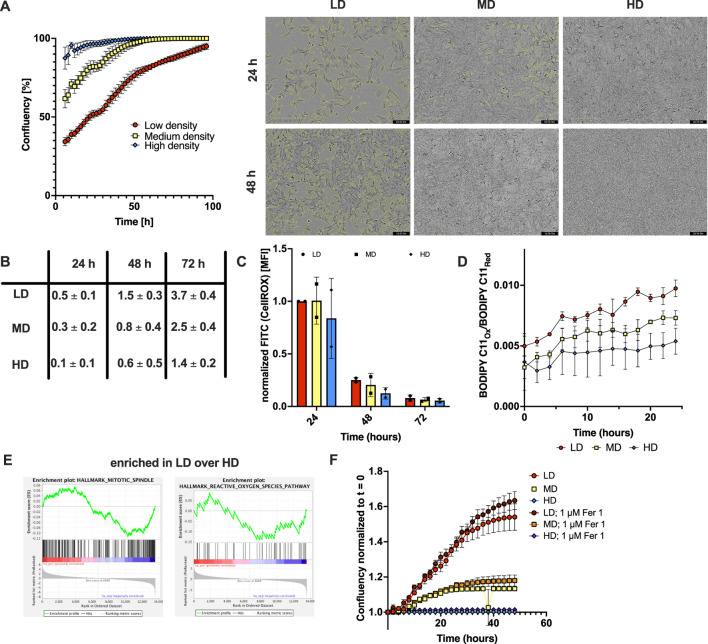
Population growth is linked to cellular ROS levels. **(A)** Confluency over time measured in MEFs seeded at 25.000 (low density, LD), 55.000 (medium density, MD) or 120.000 cells (high density, HD) per well on a 24 well plate using the Incucyte live cell imaging system. Right: brightfield images recorded at the indicated time points. Yellow margins indicate cell boundaries. **(B)** Number of population doublings occurring in MEF populations seeded at LD, MD and HD during 24, 48 and 72 h of incubation. Population doublings were calculated from the starting cell number and the cell numbers measured after the indicated time points. **(C)** Total cellular ROS measured using the CellROX dye in FACS at the indicated time points of MEF populations seeded at LD (red), MD (yellow) and HD (blue). Shown is MFI (mean fluorescence intensity) normalized to LD at 24 h within each of the replicates. **(D)** Lipid ROS over time shown as oxidized-to-reduced ratio of the STY-BODIPY dye, measured in the Incucyte live-cell imaging system at the indicated time points of MEF populations seeded at LD (red), MD (yellow) and HD (blue). **(E)** Exemplary gene sets from Gene set enrichment analysis (GSEA) are shown. GSEA hallmark Mitotic spindle pathway was significantly enriched (normalized enrichment score (NES) = -1.8, false discovery rate (FDR) q = 0.054, p-value 0.01). While the GSEA hallmark reactive oxygen species pathway was not significantly enriched its NES = −1.14 and FDR q = 0.576. **(F)** Confluency over time measured in MEFs seeded at 4,000 (low density, LD, bright and dark red), 9,000 (medium density, MD, yellow and orange) or 20.000 cells (high density, HD, blue and purple) per well on a 96 well plate in presence of DMSO or 1 µM Ferrostatin-1 (Fer-1) using the Incucyte live cell imaging system.

To test the hypothesis that cellular proliferation rates are accompanied by high metabolic generation of ROS in non-epithelial cells, we made use of mouse embryonic fibroblasts (MEFs) seeded at low (LD), middle (MD) and high cellular density (HD) into 24-well plates while each population (LD, MD, HD) received the same amount of growth medium, tracked cellular confluence over time using live cell imaging and calculated doubling times. As expected, cells seeded at HD reached confluence first, followed by MD and LD (Figure 1A). Importantly, cells seeded at HD showed an increasingly slowing rate of population doublings between 24, 48 and 72 h in comparison to cells seeded at LD or MD (Figure 1B), suggesting progressively slower proliferation rates at HD. Next, we measured levels of cellular ROS comparing LD, MD and HD cells using the fluorescent ROS tracer dye CellROX. Indeed, mean fluorescent intensity (MFI) of the tracer decreased in all three starting densities over time with increased cellular density indicative of lower levels of ROS (Figure 1C). Notably, with labile iron present, lipid ROS can arise through spontaneous Fenton reactions. Indeed, unperturbed basal cellular lipid ROS levels were highest in LD cells, with all cell populations showing a tendency towards a very slight accumulation of lipid ROS over time (Figure 1D), albeit hardly above baseline levels. To assess whether the observed changes in cellular proliferation and ROS levels were accompanied by alterations in gene expression, we performed RNA-sequencing (RNA-seq) on MEFs seeded at LD versus HD under identical culture conditions (well size and media volume). Interestingly, mere changes in growth rates resulting from these two different seeding densities was sufficient to significantly alter gene expression programmes in the two states. Interestingly, gene set enrichment analysis (GSEA) showed a significant enrichment of a mitotic spindle gene set (normalized enrichment score (NES) = -1.8, false discovery rate (FDR) q = 0.054, p-value 0.01), whereas the ROS pathway showed a non-significant tendency towards enrichment (NES = −1.14 and FDR q = 0.576) (Figure 1E), suggesting proliferative cells with high levels of ROS to be present predominantly within the LD condition. In order to test, whether proliferation-induced, low-grade built-up of lipid ROS is detrimental to cellular growth, we then measured confluence over time in all three starting densities in absence and presence of ferroptosis inhibitor Ferrostatin-1 (Fer-1), a synthetic antioxidant ferroptosis inhibitor that acts as lipophilic radical scavenger (Figure 1F) (Dixon et al., 2012). Indeed, cells in the LD condition showed a small tendency towards enhanced cellular growth upon baseline inhibition of ferroptosis. Taken together, these data indicate that population growth dynamics are strongly linked with cellular ROS levels.

### A mathematical model incorporates population growth, lipid ROS, and ferroptosis into a feedback mechanism

We developed a mathematical model to quantify the connection between population growth, lipid ROS, and ferroptosis. Our minimal model consists of three interlinked components: (i) population dynamics driven by logistic growth and ferroptosis-mediated cell death; (ii) the production of ROS as a by-product of rapid cell growth, which contributes to lipid ROS formation and, via the Fenton reaction, to lipid peroxidation (LPO); and (iii) ferroptotic cell death triggered by LPO.

**FIGURE 2:**
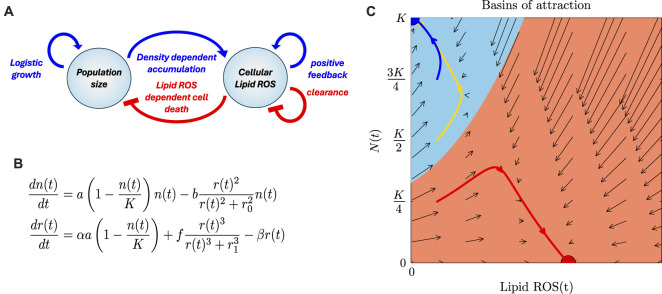
A mathematical model of the interaction between population growth, levels of lipid ROS, and ferroptosis. **(A)** Schematic overview of the feedback loop linking population dynamics and lipid ROS levels. **(B)** Mathematical model describing the feedback loop linking population dynamics and lipid ROS levels. n, population size; r, average levels of lipid ROS per cell; K, carrying capacity; a basal rate of cell divisions; b basal rate of cell death; a, ROS generation rate per cell division; b, ROS depletion rate per molecule. **(C)** Basin of attraction plot resulting from the mathematical model, where the lines represent sample trajectories starting from different seeding densities (blue–HD; yellow–MD; red–LD). Areas colored orange or blue indicate basins on attraction, i.e., starting points that will evolve either to the ferroptosis sensitive state (orange) or the insensitive state (blue). Arrows indicate the dynamics defined by the model (see Supplementary Document S1).

These three components set up a negative feedback loop, in which ROS levels emerge as a by-product of rapid cell growth, which in turn elevates lipid ROS levels, leading to increased LPO and subsequent ferroptosis, which ultimately limits further growth (Figure 2A). We focus on two variables: the population size (*n*) and the average lipid ROS level per cell (*r*) and describe their behaviour using two differential equations: Population size increases logistically toward a carrying capacity (*K*) but decreases when lipid ROS exceeds a threshold (*r*_0_) at which LPO production surpasses scavenging and repair, resulting in ferroptosis. Lipid ROS increases at a rate *a* per cell division and decreases at a rate *b* per molecule due to scavenging and degradation (Figure 2B). We also incorporated a recently described positive-feedback loop, in which lipid ROS promotes further lipid ROS production through the Fenton reaction (Co et al., 2024). The resulting system of equations predicts both the temporal dynamics and the long-term fate of the cell population.

The feedback loop linking population dynamics, lipid ROS levels, and ferroptotic cell death generates distinct cell fates that depend on the initial seeding density. Each point in Figure 2C corresponds to a lipid ROS level (*r*, x-axis) and a population size (*n*, y-axis), with arrows indicating the trajectories over time. Populations starting with low cell numbers converge on a ferroptosis-sensitive steady state characterized by high cell death and low population size (red point). In contrast, populations beginning with higher cell numbers and lower lipid ROS evolve toward a ferroptosis-resistant steady state in which the population reaches carrying capacity. The initial conditions that lead to either the sensitive or resistant states—corresponding to LD, MD, or HD seeding, or to different rates of growth-associated ROS generation—define the so-called basins of attraction for these outcomes (shaded in blue and red). The locations of these basins are parameter-dependent (see Supplementary Material).

### Seeding density determines cellular ROS levels and sensitivity to GPX4 inhibition

**FIGURE 3:**
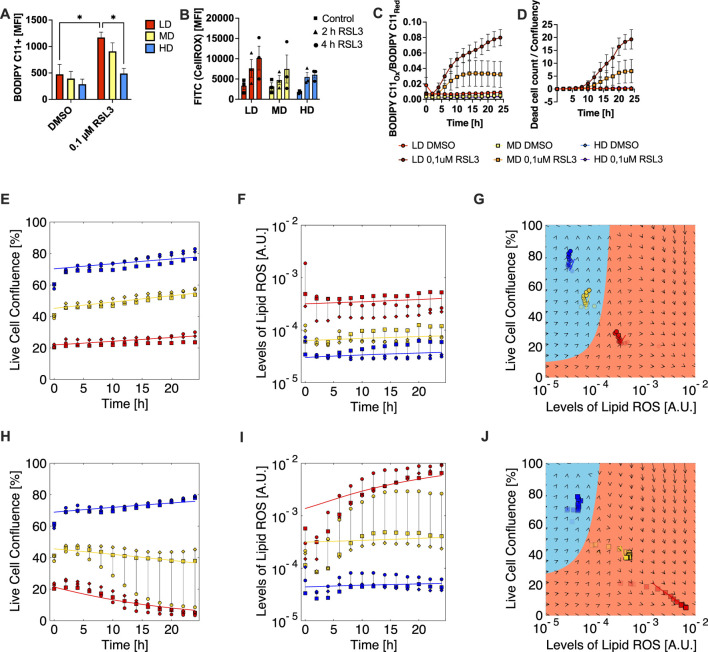
Ferroptosis sensitivity is linked with the clearance of lipid peroxidation products. **(A)** Total lipid ROS measured using Bodipy C11 dye in FACS at the indicated time points of MEF populations seeded at LD, MD and HD in 24-well plates and in presence of vehicle control (DMSO) or GPX4 inhibitor RSL3 (0.1 μM), MFI, mean fluorescence intensity. **(B)** Total cellular ROS measured using the CellROX dye in FACS at the indicated time points of MEF populations seeded at LD, MD and HD in 12-well plates and in presence of DMSO or 0.1 µM RSL3. **(C)** Lipid ROS over time shown as oxidized-to-reduced ratio of the STY-BODIPY dye, measured in the Incucyte live-cell imaging system at the indicated time points of MEF populations seeded at LD, MD and HD in 12-well plates and in presence of DMSO or 0.1 µM RSL3. **(D)** Dead cell counts over time shown as dead cell count-to-confluence ratio using t DRAQ7 dead cell dye, measured in the Incucyte live-cell imaging system at the indicated time points of MEF populations seeded at LD, MD and HD in 12-well plates and in presence of DMSO or 0.1 µM RSL3. Data in A-D are shown as mean ± SEM.; *p ≤ 0.05 (one-way Anova). **(E)** Live cell confluency of the Incucyte measurement corresponding to data shown in **(C,D)**. Colors indicate different starting population sizes (blue–HD; yellow–MD; red–LD), and differently shaped symbols represent different replicates. Data from DMSO-treated populations shown. **(F)** Levels of lipid ROS in live cells computed from the Incucyte measurements displayed in **(C,D)**, DMSO-treated population. Colors indicate different starting population sizes (blue–HD; yellow–MD; red–LD), and differently shaped symbols represent different replicates. **(G)** Basins of attractions plot showing live cell confluence versus live cell lipid ROS levels computed from **(E)** and **(F)**. Colors of symbols indicate different starting population sizes (blue–HD; yellow–MD; red–LD), for clarity only a single replicate is shown. Data from DMSO-treated populations. **(H)** Live cell confluency of the Incucyte measurement corresponding to data shown in **(C,D)**. Colors indicate different starting population sizes (blue–HD; yellow–MD; red–LD), and differently shaped symbols represent different replicates. Data from 0.1 µM RSL3-treated populations shown. **(I)** Levels of lipid ROS in live cells computed from the Incucyte measurements displayed in **(C,D)**. Colors indicate different starting population sizes (blue–HD; yellow–MD; red–LD), and differently shaped symbols represent different replicates. Data from 0.1 µM RSL3-treated populations. **(J)** Basins of attractions plot showing live cell confluence versus live cell lipid ROS levels computed from **(H)** and **(I)**. Colors of symbols indicate different starting population sizes (blue–HD; yellow–MD; red–LD), for clarity only a single replicate is shown. Data from 0.1 µM RSL3-treated populations. Parameters for the fits in **(E)**, **(F)**, **(H)**, **(I)**, and for the basins of attraction if **(G)** and **(J)** are come from least-square fitting the mathematical model to the data, see Methods and Supplementary Document S1.

To test our mathematical model experimentally, we manipulated the parameters characterizing the feedback loop between population dynamics, ROS levels and ferroptosis. We started by interfering with GPX4 activity using the small molecule inhibitor RSL3 (Yang and Stockwell, 2008). Since constitutive GPX4 activity leads to clearance of LPO products, GPX4 inhibition leads to increased levels of LPO. Excessive LPO leads to membrane destabilization, permeabilization (Pedrera et al., 2020) and ferroptotic cell death (Bebber et al., 2020; Conrad and Pratt, 2019). Interestingly, RSL3 treatment led to increased detection of both lipid ROS and total ROS as the seeding cell density was decreased from HD to LD (Figures 3A,B). Moreover, lipid ROS increased over time in a strict density-dependent manner upon RSL3-treatment (Figure 3C). As a result of this, cells seeded at LD and MD but not HD underwent cell death upon RSL3 treatment (Figure 3D). These outcomes match the predictions of our mathematical model: We fit the model parameters to the experimental time courses of confluence and lipid ROS levels, resulting in the matching curves comparing the results of the model and experiments shown in Figures 3E–J, both for the control without RSL3 and the experiments with GPX4 inhibition using RSL3. As expected, we find the inferred lipid ROS threshold parameter *r*_*0*_ reduced under RSL3 relative to the control, since less lipid ROS can induce ferroptosis without GPX4 activity. This leads to a shift in the basins of attraction specifying cell fate (Figures 3G,J): under RSL3, cell death is triggered already at lower lipid ROS rates due to reduced LPO clearance. This reduces the population size, and leads to a faster turnover of residual cells, and (averaged over the surviving cells) increased levels of lipid ROS, model predictions which are all borne out experimentally.

### Ferroptosis sensitivity is restricted by growth space and not limited RSL3 availability per cell

**FIGURE 4:**
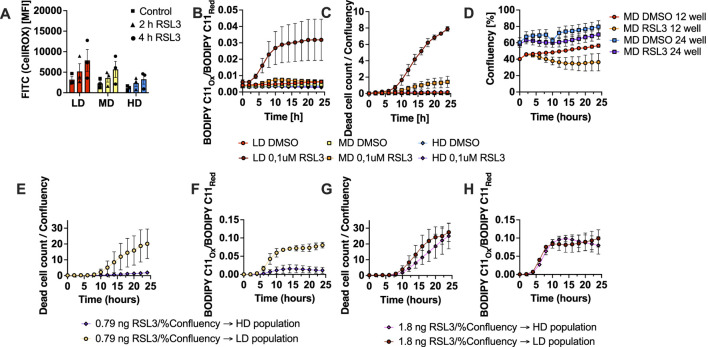
Ferroptosis sensitivity is restricted by growth space. **(A)** Total cellular ROS measured using the CellROX dye in FACS at the indicated time points of MEF populations seeded at LD (red), MD (yellow) and HD (blue) in 24-well plates in presence of DMSO or 0.1 µM RSL3. **(B)** Lipid ROS over time shown as oxidized-to-reduced ratio of the STY-BODIPY dye, measured in the Incucyte live-cell imaging system at the indicated time points of MEF populations seeded at LD (red, bright red), MD (yellow, orange) and HD (blue, purple) in 24-well plates presence of DMSO or 0.1 µM RSL3. **(C)** Dead cell counts over time shown as dead cell count-to-confluence ratio using the DRAQ7 dead cell dye, measured in the Incucyte live-cell imaging system at the indicated time points of MEF populations seeded at LD (red, bright red), MD (yellow, orange) and HD (blue, purple) in 24-well plates in presence of DMSO or 0.1 µM RSL3. **(D)** Confluency over time of the Incucyte measurements at the indicated time points of MEF populations seeded at MD in 12-well and 24-well plates in presence of DMSO or 0.1 µM RSL3. Confluency data in 12-well plates is taken from the measurements displayed in Figure 3D. **(E)** Dead cell counts over time shown as dead cell count-to-confluence ratio using the DRAQ7 dead cell dye, measured in duplicates using the Incucyte live-cell imaging system at the indicated time points of MEF populations seeded at LD and HD in 24-well plates in presence of DMSO or 0.79 ng/percent confluence RSL3 (HD dose, resistant condition). **(F)** Lipid ROS over time shown as oxidized-to-reduced ratio of the STY-BODIPY dye, measured in duplicates using the Incucyte live-cell imaging system at the indicated time points of MEF populations seeded at LD and HD in 24-well plates in presence of DMSO or 0.79 ng/percent confluence RSL3 (HD dose, resistant condition). **(G)** Dead cell counts over time shown as dead cell count-to-confluence ratio using the DRAQ7 dead cell dye, measured in duplicates using the Incucyte live-cell imaging system at the indicated time points of MEF populations seeded at LD and HD in 24-well plates in presence of DMSO or 1.8 ng/percent confluence (LD dose, sensitive condition). **(H)** Lipid ROS over time shown as oxidized-to-reduced ratio of the STY-BODIPY dye, measured in duplicates using the Incucyte live-cell imaging system at the indicated time points of MEF populations seeded at LD and HD in 24-well plates in presence of DMSO or 1.8 ng/percent confluence (LD dose, sensitive condition). All data are shown as mean ± SEM.

To test for the possibility that in the LD condition individual cells receive relatively higher doses of RSL3 leading to increased induction of ferroptosis, we repeated RSL3 treatments of cells seeded at LD, MD and HD comparing growth in 24 well versus 12 well plates while using the same medium volume and RSL3 concentration (per volume), thereby keeping RSL3 concentration per cell constant. By doing this, only the growth space, i.e., relative confluency of any given cell number varied between the 12- and 24-well condition. Cells grown in 24-well plates at all three starting densities showed a smaller induction of general ROS upon treatment with RSL3 (Figure 4A) in comparison to the 12-well conditions (Figure 3B). Importantly, the MD condition seeded in 24-well plates was much more resistant to lipid ROS and cell death (Figures 4B,C) than the same cell number treated with the same drug concentration per cell in a 12-well format with more growth space (Figures 3C,D). Moreover, cultivation under restricted space (24-well) led to unperturbed continued growth of MD cells while confluency of MD cells was decreased under RSL3 treatment with unrestricted space (Figure 4D). These data support a role of high cellular confluence that causes increased ferroptosis resistance and decreased levels of ROS and lipid ROS. In our mathematical model this mechanism emerges from the density-dependent rate of cell divisions, and a rate of ROS production that is proportional to the rate of cell divisions. To further test this hypothesis experimentally, we next calculated the effective dose of RSL3/percent confluence in various seeding densities as in Figures 3A–J and Figures 4A–D, which was approximately 1.8 ng/percent confluence for LD (LD dose) and 0.79 ng/percent confluence for HD (HD dose). We then treated cells seeded at LD and HD with both, the LD and HD dose and recorded dead cell counts and lipid ROS over time. Strikingly, cells seeded at LD and treated with the HD dose (resistant condition in Figures 3, 4) showed accumulation of dead cells over time (Figure 4E), which was preceded by accumulation of lipid ROS (Figure 4F). Again, populations seeded at HD did not react to the HD dose, again showing that resistance to RSL3, in our setup could not be explained by availability of RSL3 per cell. Conversely, treatment of cell seeded at LD and HD with the LD dose (effectively increasing the dose/cell in the HD seeing condition) indeed led to accumulation of comparable amounts of lipid ROS in both populations (Figure 4H). The resulting accumulation of dead cell counts, however, was delayed in the HD condition (Figure 4G). In the mathematical model, the increased GPX4 inhibition changes the available long-term fates, leaving cell death as the only stable state at high RSL3 doses. As a result, a density-dependent ferroptosis susceptibility can only be observed within a finite range of GPX4 inhibition. Together these data indicate that while lower relative per cell doses of RSL3 contribute to cellular resistance at HD, high cellular confluence independently and in addition lowers cellular ROS levels and ferroptosis sensitivity.

### Ferroptosis sensitivity of growing populations are determined by OXPHOS-derived ROS levels

**FIGURE 5:**
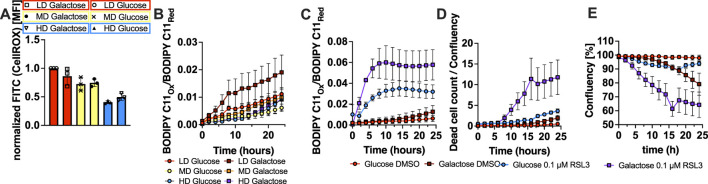
Ferroptosis sensitivity of growing populations are determined by OXPHOS-derived ROS levels. **(A)** Total cellular ROS measured using the CellROX dye in FACS at the indicated time points of MEF populations seeded at LD, MD and HD in galactose or glucose medium and in presence of DMSO or 0.1 µM RSL3. **(B)** Lipid ROS over time shown as oxidized-to-reduced ratio of the STY-BODIPY dye, measured in the Incucyte live-cell imaging system at the indicated time points of MEF populations seeded at LD, MD and HD in galactose or glucose medium **(C)** Lipid ROS over time shown as oxidized-to-reduced ratio of the STY-BODIPY dye, measured in the Incucyte live-cell imaging system at the indicated time points and in presence of DMSO or 0.1 µM RSL3 at HD. **(D)** Dead cell counts over time shown as dead cell count-to-confluence ratio using the DRAQ7 dead cell dye, measured in the Incucyte live-cell imaging system at the indicated time points of MEF populations seeded at HD in glucose or galactose medium in presence of DMSO or 0.1 µM RSL3. **(E)** Confluency over time data from measurements displayed in **(D)**. All data are shown as mean ± SEM.

Next, we aimed to experimentally increase the rate of ROS accumulation per cell division. To this end, we made use of the principle that cells generating energy equivalents in the form of ATP can do so via OXPHOS or through glycolysis (Greene et al., 2022). While the former process is more efficient in generating ATP, this efficiency comes at the cost of electron leakage from the respiratory chain in the mitochondria leading to a high rate of ROS generation (Nolfi-Donegan et al., 2020). Experimentally, cells can be forced to predominantly use OXPHOS for ATP generation by replacing glucose with galactose in growth media leading to increased ROS which should be uncoupled from cell confluency (Aguer et al., 2011). To achieve this, MEF culture media were selectively depleted and repleted for glucose or galactose in glucose-free medium supplemented with insulin-transferrin-selenium (ITS+) instead of calf serum as it is free of glucose. Notably, ITS + supplementation is vital for ferroptosis to proceed as selenium is rate-limiting for GPX4 translation and transferrin allows for uptake of iron and is thereby equally crucial for ferroptosis (Gao et al., 2015). Indeed, growing cells in glucose-free medium replenished with galactose but not glucose led to slightly increased ROS in cells grown at HD condition and increased lipid ROS in all three starting densities in comparison to glucose supplemented cells within 24 h (Figures 5A,B). In line with the mathematical model, the increase in lipid ROS in the galactose condition also led to increased lipid ROS under RSL3 treatment, resulting in strong sensitization to ferroptotic cell death in galactose-compared to glucose-treated cells at HD (Figures 5C–E). These data together with our data testing cellular growth space indicate that the inhibitory effect of high population density on ferroptosis cell fate is caused by low metabolic levels of cellular ROS and not cellular proximity.

## Discussion

By integrating mathematical modelling with experimental data, we provide evidence that cellular population dynamics, metabolic ROS production, and ferroptosis sensitivity are interlinked in a feedback loop. These results have important implications for physiological scenarios where cellular growth space or metabolism affects ROS levels, e.g., within tumours. Additionally, our results have implications for the design and control of *in vitro* studies testing ferroptosis sensitivity in fast-growing cell populations such as cancer cell lines. The modelling presented within this study focuses on the interplay between ROS, lipid ROS and ferroptosis induction, while ROS has been implicated in different kinds of cell death, including apoptosis, necroptosis and autophagy (Villalpando-Rodriguez and Gibson, 2021). Importantly, the cellular states that decide which pathway is triggered as well as their interplay in the context of high ROS levels remains poorly understood (Villalpando-Rodriguez and Gibson, 2021). Specifically, we show that low-density (LD) cultures exhibit faster proliferation, elevated ROS generation and greater sensitivity to RSL3-induced ferroptosis in comparison to medium (MD)- and high-density (HD) cultures. Populations at all starting densities showed decreasing levels of general ROS over time, in line with previous studies in fibroblasts (Burdon et al., 1995) and mesenchymal stem cells (Lyublinskaya et al., 2015). In contrast, lipid ROS in all three populations showed a faint tendency towards increasing over time, yet, remained at baseline value in comparison to RSL3-treated populations. General ROS is very short-lived (Juan et al., 2021), and CellROX-stainings were done via FACS measurements, consequently, cellular ROS reported in this study represent intracellular ROS levels at the time of the experiment. In comparison, lipid ROS data were recorded via live-cell imaging. Given that BODIPY-probes can be used to track lipid ROS over hours (Roeck et al., 2025; Siejak et al., 2023), lipid ROS data reported herein represent accumulated lipid ROS over time, resulting in lipid ROS levels not changing despite amelioration of general ROS levels. Despite baseline lipid ROS levels in untreated populations only showing a small trend towards decreasing with starting density, we also tested whether population growths could be enhanced in any of the three densities by inhibition of ferroptosis using Fer-1, finding a small tendency towards enhanced cellular growth upon inhibition of ferroptosis in the LD condition.

Comparing same sized cell populations with different growth spaces (12 vs. 24 well plates) we here hypothesized that cellular confluence and not limited drug availability per cell causes changes in ferroptosis resistance. Yet, our data show that limited drug availability per cell also promote resistance at HD in addition to limited growth space. These findings present an important limitation of our model in predicting ferroptosis that warrants further investigation and clarification of the underlying cellular dynamics. Given that ferroptosis may spread in trigger waves (Co et al., 2024), it is tempting to speculate whether in HD conditions treated with a higher per-cell LD dose of RSL3, ferroptosis was triggered in a small fraction of cells (e.g., from cells at the outer rim of the well with lower density) and subsequently spread throughout the population, causing the observed delay.

Importantly, while controlling for drug availability, use of 12 vs. 24 well plates may also have altered the partial pressure of oxygen (pO_2_) during cell culture, resulting from differently sized meniscus effects (Tan et al., 2024). While pO_2_ during the experiments carried out in this study has not been determined, it is very well known that hypoxia may regulate ferroptosis in different ways, which may result in inhibition or induction of ferroptosis (reviewed elsewhere (Zheng et al., 2023)). The inverse relationship between cellular density and sensitivity to ROS was first established by a study in human fibroblasts (Kim et al., 2012). While the concept of ferroptosis was unknown at the time, more recent studies have provided evidence for a direct link between cellular density and ferroptosis sensitivity. A specific cell state in glioma in which constitutive active NOTCH signalling increases mitochondrial ROS and ferroptosis sensitivity has been reported (Banu et al., 2024). Interestingly, epithelial cancer cell lines were shown to restrict ferroptosis via E-Cadherin-mediated YAP1 sequestration at high cellular densities and cell-to-cell contact (Wu et al., 2019). Moreover, low cell density was shown to render breast cancer cell lines ferroptosis sensitive due to elevated PUFA integration into their lipidomes (Panzilius et al., 2019).

Furthermore, the use of galactose to force OXPHOS (Aguer et al., 2011)—a known source of ROS (Labuschagne et al., 2019)—revealed that metabolic context, rather than density alone, is a key determinant of ferroptosis susceptibility. Interestingly, oxygen consumption in cell populations has long been known to decrease markedly upon reaching confluency (Bereiter-Hahn et al., 1998; Jorjani and Ozturk, 1999). More recent studies have shown that mitochondrial activity decreases with cellular density in epithelial cells (Thurakkal et al., 2023), while oxygen consumption in 3D tissue models using HepG2 cells is markedly increased at lower densities (Magliaro et al., 2019), and proliferating fibroblasts show an increase of OXPHOS by 81% in comparison to quiescent fibroblasts (Yao et al., 2019), a cell state that is commonly induced by high density conditions (Fan and Meyer, 2021). Taken together with our findings, these insights underscore a potential role of OXPHOS in the regulation of ferroptosis sensitivity. Whether the increase in ferroptosis sensitivity in low density and galactose supplemented cell populations observed in this study are only driven by OXPHOS upregulation and hence share a common underlying mechanism remains to be clarified in further studies. Of note, the elicitation of OXPHOS in proliferating fibroblast population is driven by mitochondrial fusion (Yao et al., 2019), a process by which cells adapt to metabolic challenges such as fasting (Lee et al., 2014), whereas quiescent cells show signs of mitochondrial fragmentation (Yao et al., 2019). In light of our findings linking density-dependent proliferation to OXPHOS and thereby ferroptosis sensitivity, this supports and refines earlier studies indicating that mitochondrial ROS contribute to lipid peroxidation and ferroptosis in specific contexts (Gaschler et al., 2018; Nolfi-Donegan et al., 2020). Apart from ferroptosis, mitochondrial ROS can also trigger intrinsic apoptosis via cytochrome C release (reviewed elsewhere (El-Osta and Circu, 2016)). Importantly, the specific cellular context under which apoptosis or ferroptosis is induced remain to be closer defined in follow-up studies in order to expand and improve our model in predicting ferroptosis sensitivity of growing populations. Our findings show that seeding density is actively linked with ferroptosis sensitivity through a negative feedback mechanism involving ROS dynamics and proliferation rates. Importantly, our mathematical modelling aligns with the emerging understanding of ferroptosis regulation as a systems-level process with multiple feedback loops (Co et al., 2024). While prior models have considered the role of iron, lipid metabolism, and antioxidant systems (Konstorum et al., 2020), our model uniquely incorporates population growth dynamics, revealing bistable outcomes (ferroptosis-sensitive vs. -resistant states) depending on initial conditions such as cell density and ROS levels. This prediction was experimentally validated using scaled culture formats (12- vs. 24-well plates), ruling out drug dilution as a confounding factor and emphasizing that cell density and growth-induced ROS accumulation drive the feedback loop. This model explains the seemingly paradoxical observation that low-density cell populations—unrestricted by spatial constraints—can initiate ferroptosis. This phenomenon arises from the accumulation of reactive oxygen species (ROS) generated during rapid expansion. In principle, such a mechanism could affect any context of accelerated cellular growth, where instead of establishing a large population, the cells undergo population collapse due to ferroptotic cell death. Central to our model is a negative feedback loop: rapid cell divisions lead to increased ROS production, which induces lipid peroxidation and ultimately triggers ferroptosis. This dynamic gives rise to bistable behaviour, with one state marked by low proliferation, minimal ROS levels, and absence of ferroptosis, and the other characterized by high proliferation and turnover, elevated ROS levels, and ferroptotic cell death.

Together, our findings position cell density as both a predictor and regulator of ferroptosis susceptibility, mediated through ROS and metabolic activity. This has broad implications for tissue homeostasis, cancer biology, and therapy resistance, particularly in light of recent interest in exploiting ferroptosis as a therapeutic vulnerability in proliferative and therapy-resistant tumour populations (Hassannia et al., 2019). By integrating experimental data with predictive modelling, our study adds a quantitative framework to the qualitative observations previously reported, offering a deeper mechanistic understanding of how proliferative states and metabolic stress shape ferroptotic outcomes.

## Data Availability

The raw data supporting the conclusions of this article will be made available by the authors, without undue reservation.
